# Optimal Finite Difference Angular Velocity Estimation for Spacecraft

**DOI:** 10.1007/s40295-026-00570-6

**Published:** 2026-02-19

**Authors:** Jack P. Leo, John P. Enright

**Affiliations:** Department of Aerospace Engineering, Toronto Metropolitan University, 350 Victoria St, Toronto, ON M5B 2K3 Canada

**Keywords:** Angular velocity estimation, Error covariance, Finite difference approximation, Spacecraft attitude estimation, Star trackers

## Abstract

This paper presents a practical, computationally efficient approach to spacecraft angular velocity estimation using the finite difference (FD) differentiation of star tracker attitude measurements. Intended for gyro-free applications such as within the star tracker processors themselves, this technique is not reliant on external sensors. Although prior studies have proposed similar finite difference techniques, this study provides a more accurate and rigorous model of angular velocity covariance. Additionally, we derive an analytical model of optimal measurement timing to balance noise and bias in the finite difference estimates. A series of simulations validates the revised covariance models and benchmarks the performance of the finite difference rate estimator against a conventional Multiplicative Extended Kalman Filter (MEKF). Although the FD estimates show significant latency-induced bias, the standard deviation of the measurements are improved by 40% or more compared to the MEKF.

## Introduction

Although star trackers are a convenient single instrument source of precise three-axis attitude knowledge [1], these sensors are most effective when combined with an accurate measure of the host spacecraft’s angular velocity. Rolling shutter detectors are particularly susceptible to inaccuracy due to rate uncertainty [2], but even global shutter detectors can benefit from better rate knowledge, particularly during agile maneuvers. This study explores techniques that can be used to improve the angular velocity estimates available for star tracker processing.

This study focuses on improving the quality of angular velocity estimates generated within the star tracker itself, specifically targeting factors such as latency, accuracy, and precision. From a systems-level perspective, this is not a particularly challenging prospect; the attitude determination and control system (ADCS) can merely fuse the measurements from a star tracker and a high-quality rate-gyro. However, there are some subtle complications if our focus shifts to the star tracker instrument alone. For the star tracker designer, relying on external gyro measurements can lead to complex electrical and data interfaces. Conversely, directly integrating a rate gyro into the star tracker itself is not always straightforward either. Small, MEMS-based gyros are the most attractive components to minimize the impact on instrument mass, volume, and power requirements, yet the drift and bias characteristics of these components are often quite poor [3]. Thus, this study focuses solely on angular velocity estimates derived from star observations.

Angular rate estimates derived from star trackers generally follow one of two approaches [4]. *Estimation* methods use different types of recursive filters to generate rate estimates from star vectors or quaternion measurements, relying on system dynamics (i.e., rotational kinematics) to update the rate states; *derivative* methods explicitly relate the changing attitude to the angular velocity.

Estimation methods are widely used and are generally based on one of the many variants of Kalman Filters (KF). Critchley-Marrows et al. [5], evaluates an additive and multiplicative extended Kalman filter for both attitude and rate information. Liu et al. [6], use an adaptive KF to estimate attitude measurement covariance, online. Most researchers assume simple measurement models, but others, such as Ning et al. [7], use the shape of the smeared star images as an additional source of information.

Derivative techniques generally relate the changing attitude representation—e.g., quaternions, rotation matrices, (modified) Rodrigues parameters, etc.—to the angular velocity and then generate rate estimates accordingly. Although these techniques may not be optimal in a maximum likelihood or minimum variance sense, they generally have very modest computational requirements and rarely suffer from convergence difficulty. Crassidis’ approach [8], provides a least squares estimate of the angular velocity between star tracker frames without needing to identify the stars directly. Jo et al. [9], uses body vector measurements in their Wahba’s problem-based method and gives an expression for the covariance of the angular velocity estimate.

One primary drawback of derivative techniques is the introduction of high-frequency noise due to the finite difference (FD) differentiation approximation [4], a phenomenon exacerbated by short sampling periods (e.g., high update rates). Extending the sample period can reduce noise, but this can introduce latency induced bias in the calculated body rates especially when the angular velocity is not constant. Additional kinematic information, such as angular acceleration, can reduce the impact of this latency but adds complexity to the sensor data interface. The error characteristics of these FD estimates are crucial for understanding the overall accuracy and behaviors of the estimates.

Covariance analysis can be used to relate errors in vector and orientation measurements to errors in the estimated angular velocity. However, the existing literature on this topic is rather limited. Many studies on angular velocity estimation fuse measurements from star trackers, sun sensors, magnetometers, and other attitude instruments [10–19]. Others estimate angular velocity using optical flow derived from star images [20–23]. However, these works provide very limited covariance analysis and typically include only enough detail to allow recursive estimators to incorporate the raw measurement covariance.

Despite the limited treatment in the existing literature, some publications do provide predictions of the angular velocity error covariance. Both Crassidis [8], and Jo et al. [9], derive expressions that characterize the angular velocity error covariance based on body vector measurements. These formulations build on the orientation error covariance matrix expression originally derived by Shuster [24]. The 2017 work by Jo et al. [25] builds upon their covariance analysis [9], providing an optimal time delay between measurements. Other methods, such as the QuateRA algorithm, propose a batch estimation method to estimate the spin-axis direction (SAD) and the angular velocity magnitude (AVM) using orientation measurements. The algorithm employs a recursive method based on a MEKF formulation and the Fisher Information Matrix approach to estimate the angular velocity error covariance [26]. Kaki et al. [27], addresses the limitations of the recursive method by developing a batch estimation approach to estimate the SAD and its corresponding covariance. The QuateRA+ algorithm combines the original QuateRA algorithm with the SAD covariance batch estimation method to provide a comprehensive batch estimation approach for the SAD, AVM, and the overall angular velocity vector, along with their respective covariances [28]. QuateRA+ also addresses the limitations of the proposed method by Kaki et al. [27], by using quaternions rather than Euler angles and rotated unit vector sets, thereby simplifying the method and enhancing computational efficiency. However, these studies limit their closed-form expressions for the angular velocity error covariance to the isotropic measurement noise assumption. While this assumption is reasonable, it is not always applicable, especially for star tracker attitude measurements.

This study presents an optimized and comprehensive framework for finite difference angular velocity determination. To minimize implementation complexity, we do not include a spacecraft dynamics model, instead relying solely on the perceived motion of stars within the field of view. The specific contributions of this study include:

1. The development of a computationally inexpensive technique for calculating angular velocity from a sequence of attitude measurements.
2. The derivation of a new closed-form covariance model for the resulting angular velocity estimates that accounts for the anisotropic nature of star tracker attitude errors. This model extends the covariance analyses of previous studies [8, 9, 26–28], which rely on various isotropic attitude noise assumptions. In particular, Almeida et al. [26], acknowledges that their isotropic noise assumptions are not always true, especially with star tracker attitude measurements. Unlike prior works, [27, 28], we only express the error covariance of the angular velocity vector, as it can be easily incorporated into higher level processing.
3. The development of an analytical expression for the optimal time step between measurements for the FD method, derived based on the improved covariance model and under the assumption of constant angular acceleration.

This paper is organized into five major sections. Section 2 provides the mathematical framework for the estimation of angular velocity and its covariances. Section 3 builds upon this framework to demonstrate how the process can be optimized to minimize errors in the estimates for a constant angular acceleration. Section 4 validates these optimizations for an *x*-axis rotation and an arbitrary axis of rotation, and compares the FD performance with that of a KF-based estimator.

## Mathematics of Rate Estimation

This section examines the attitude measurements from star trackers and the formulation of rate estimation using those measurements. Next, we examine popular covariance models for these estimates and identify their limitations when dealing with a star tracker’s anisotropic error and we derive an improved expression that is a better match to these sensors.

### Rate Estimation

For this analysis, we assume that the spacecraft rotational motion is represented by orientation, expressed as scalar first quaternions, ${\textbf{q}}$, and angular velocity, ${\boldsymbol{\omega }}$,

1 $$\begin{aligned} {\textbf{q}} = \begin{bmatrix} q_{s} \\ {\textbf{q}}_{v} \end{bmatrix} = \begin{bmatrix} q_{0} \\ q_{1} \\ q_{2} \\ q_{3} \end{bmatrix}, \ {\boldsymbol{\omega }} = \begin{bmatrix} \omega _{x} \\ \omega _{y} \\ \omega _{z} \end{bmatrix} \end{aligned}$$

Star trackers sample orientation at discrete times, ${\textbf{q}}\left( t_{k}\right)$, and these measurements are corrupted by noise. We generally express this as

2 $$\begin{aligned} \widetilde{{\textbf{q}}}\left( t_{k}\right) = {\boldsymbol{\delta }}{\textbf{q}}\left( t_{k}\right) \otimes {\textbf{q}}\left( t_{k}\right) \end{aligned}$$

**Fig. 1:**
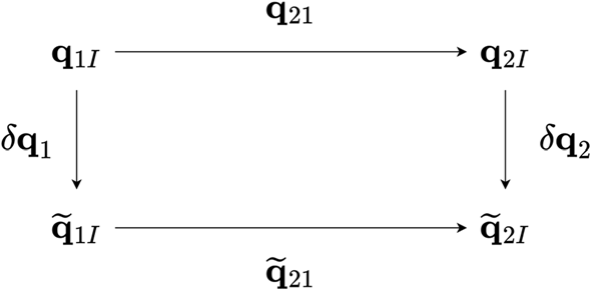
Quaternion relationships between relative quaternions and measurement noise

In our notation, we take the sense of the rotation as specifying the rotation into the frame of the sensor, i.e., ${\textbf{q}}_{SI}$ and that the error is composed of an additional rotation relative to the true frame. If we consider two quaternion measurements taken at times $t_{1}$ and $t_{2}$, then we have the following relationships presented in Fig. 1.

From these sets of dependencies we can convert the relative quaternion, ${\textbf{q}}_{21}$, between any two absolute quaternions, ${\textbf{q}}_{1I}$ and ${\textbf{q}}_{2I}$. We can also see how noise, ${\boldsymbol{\delta }}{\textbf{q}}_{1}$ and ${\boldsymbol{\delta }}{\textbf{q}}_{2}$, can corrupt the measurements. The relative quaternion is related to the absolute quaternions by

3 $$\begin{aligned} {\textbf{q}}_{21}^{} = {\textbf{q}}_{2I}^{} \otimes {\textbf{q}}_{1I}^{-1} \end{aligned}$$

where ${\textbf{q}}_{21}$ represents the rotation from frame-1 to frame-2 and ${\textbf{q}}_{2I}^{}$ and ${\textbf{q}}_{1I}^{}$ represents the rotation from the inertial frame to frame-2 and frame-1, respectively. We note in passing that, whilst we have assumed that ${\textbf{q}}_{21}$ has been calculated from two sequential inertial attitude measurements, it could also be computed using relative motion between $t_{1}$ and $t_{2}$. Thus, the corresponding star vector observations between the two frames can yield ${\textbf{q}}_{21}$ without a successful catalog match or absolute attitude fix. This allows this technique to operate similarly to the method described by Crassidis [8].

The equivalent Direction Cosine Matrix (DCM) for any quaternion can be calculated using the following common formula:

4 $$\begin{aligned} {\textbf{C}}_{21} = {\textbf{C}}\left( {\textbf{q}}_{21}^{}\right) = \left( q_{s}^{2}- {\textbf{q}}_{v}^{T}{\textbf{q}}_{v}^{}\right) {\textbf{I}} + 2{\textbf{q}}_{v}^{}{\textbf{q}}_{v}^{T} - 2q_{s}^{}{\textbf{q}}_{v}^{\wedge } \end{aligned}$$

where $\left( \cdot \right) ^{\wedge }$ is the skew symmetric cross-matrix operator defined commonly in literature. We make use of the notation $\left( \cdot \right) ^{\vee }$ to indicate the inverse of the cross-matrix operation, i.e., converting the skew symmetric cross-matrix back into the source vector. The last thing to note about DCMs is the use of the matrix exponential and logarithm functions to convert between SO(3) notations (DCMs) and $\mathfrak {so(3)}$, the corresponding cross-matrix, the details of which can be found in the literature [29]. In short, ${\textbf{C}}\in$ SO(3), ${\boldsymbol{\phi }}^{\wedge }\in \mathfrak {so(3)}$ and ${\boldsymbol{\phi }}\in \mathbb {R}_{3}$. The vector ${\boldsymbol{\phi }}$ is the axis-angle representation of the rotation. It is defined as

5 $$\begin{aligned} {\boldsymbol{\phi }} = \phi {\textbf{a}} \end{aligned}$$

where $\phi$ is the scalar total angle of rotation and $${\textbf{a}} = \begin{bmatrix} a_{x}&a_{y}&a_{z} \end{bmatrix}^{T}$$ is the axis of rotation.

Given the relationship between axis-angle form and DCMs, they can be exploited to estimate the angular velocity of the rotation. When the angular motion is slow or the measurements are closely spaced in time, we can approximate the angular velocity from the difference in orientation as

6 $$\begin{aligned} \widehat{{\boldsymbol{\omega }}}_{2}^{2/1} \approx \frac{\ln \left( {\textbf{C}}_{21}\right) ^{\vee }}{\Delta t} = \frac{{\boldsymbol{\phi }}_{21}}{\Delta t} \end{aligned}$$

where $\widehat{{\boldsymbol{\omega }}}_{2}^{2/1}$ is the estimate of the angular velocity of frame-2 relative to frame-1 in frame-2 and $\Delta t = t_2 - t_1$ is the time interval between the attitude measurements. It should be noted that the angular velocity estimate obtained from the above equation is the average angular velocity over that time interval. Furthermore, because the measurements we obtain from the star tracker are corrupted by noise, the estimate itself will also be corrupted. It is crucial that $\widetilde{{\boldsymbol{\omega }}}\Delta t < \pi$ even with the presence of noise to avoid inverting the rotation axis. Given the discussed relationships, it is more practical to convert the relative quaternion, (3), straight into axis-angle form using

7 $$\begin{aligned} \phi _{21}&= 2\arccos \left( q_{s}\right) \end{aligned}$$

8 $$\begin{aligned} {\textbf{a}}_{21}&= \frac{{\textbf{q}}_{v}}{\sin \left( \frac{\phi _{21}}{2}\right) } \end{aligned}$$

### Current Angular Velocity Error Covariance Model

Jo et al. [25], approximates the angular velocity error covariance, ${\textbf{R}}_{\omega }$, as

9 $$\begin{aligned} {\textbf{R}}_{\omega } \approx \frac{1}{\Delta t^2} \left( {\textbf{R}}_{1}+{\textbf{R}}_{2} \right) \end{aligned}$$

where ${\textbf{R}}_{1}$ and ${\textbf{R}}_{2}$ are the noise covariance matrix of the attitude measurements at $t_{1}$ and $t_{2}$. The diagonal terms of ${\textbf{R}}_{1}$ and ${\textbf{R}}_{2}$ represent the measurement noise of the star tracker’s principal axes. The boresight axis (*z*-axis) of the star tracker frame is the viewing direction of the sensor and the cross-boresight axes are the *x*- and *y*-axes of the frame.

The approximation of (9) is reasonable when the noise covariance is isotropic, i.e., ${\textbf{R}}_{1} = {\textbf{R}}_{2} = \sigma ^2 {\textbf{I}}_{3\times 3}$, where $\sigma ^2$ is the covariance of the attitude noise. However, this approximation fails when ${\textbf{R}}_{1}$ and ${\textbf{R}}_{2}$ are anisotropic and the diagonal elements $\sigma _i^2$ (where $i = x,\ y,\text { and } z$) are different for each axis. Star trackers often do not exhibit isotropic orientation errors, the uncertainty of the boresight rotation is often much worse than the cross-boresight errors. This leads to inaccurate estimates of the angular velocity covariance as angular velocity increases.

**Fig. 2:**
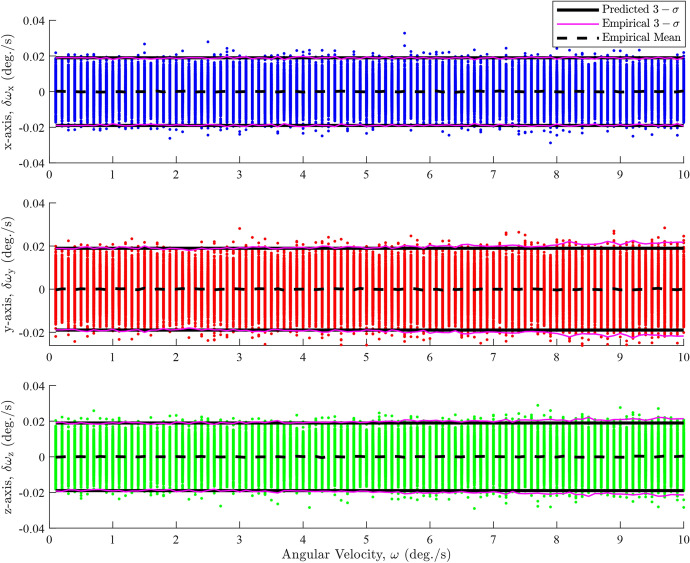
Constant angular velocity error dependencies on rate for isotropic attitude noise

Figure 2 shows the angular velocity errors on each axis for a pure *x*-axis rotation with isotropic attitude noise and constant angular velocity. The data are obtained from noisy estimates of spacecraft orientation using (6) with a fixed $\Delta t = 10 \ \text {s}$ which creates the vertical distribution of points for each angular velocity value. The isotropic attitude noise used in the simulation is $\sigma ^2 = 2\times 10^{-3} \ \text {degree}^{2}$.

**Fig. 3:**
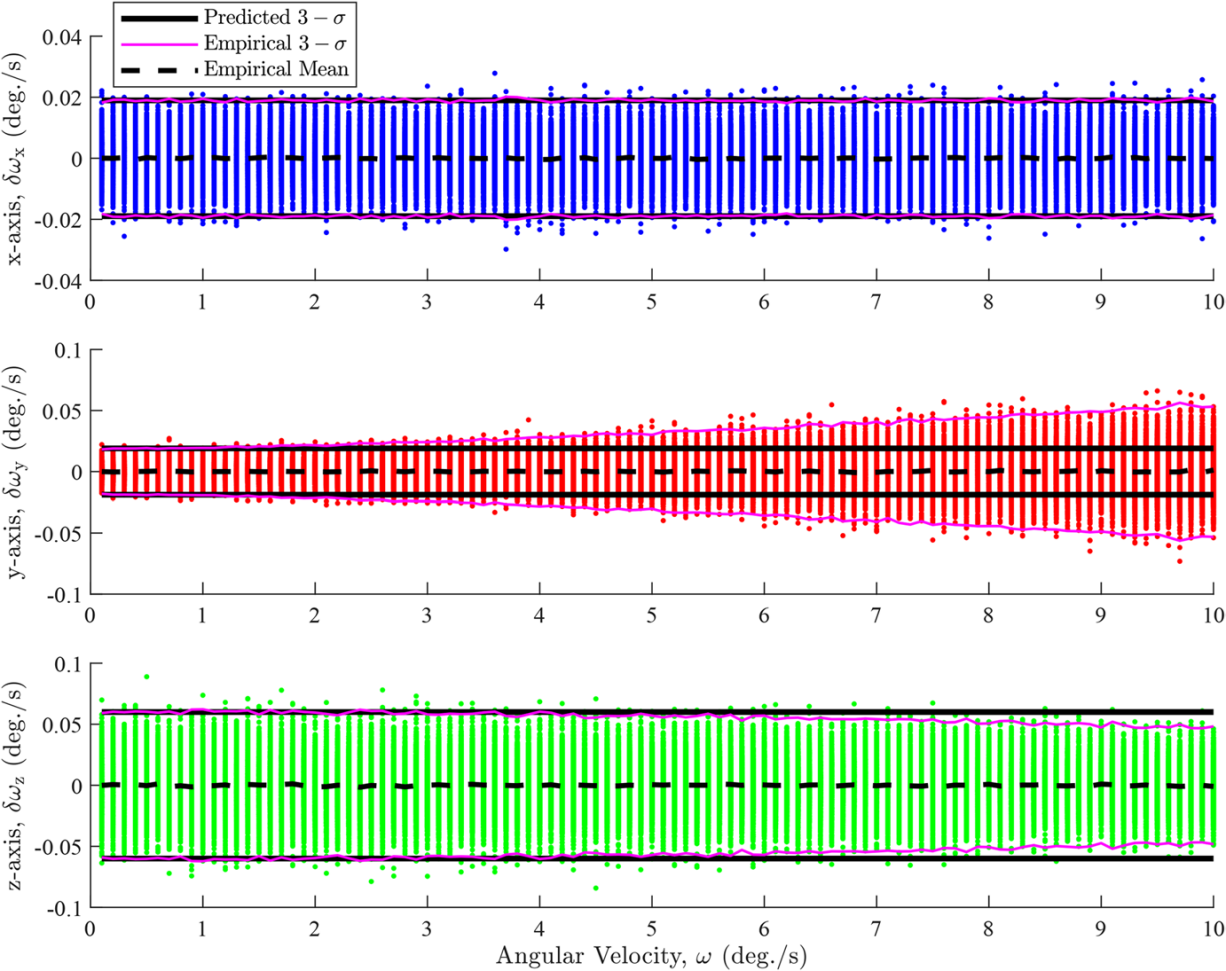
Constant angular velocity error dependencies on rate for anisotropic attitude noise

Figure 2 demonstrates that the predicted $3-\sigma$ intervals, derived from the diagonal elements of (9), generally align with the empirical (sample) $3-\sigma$ intervals. A closer inspection reveals that the empirical and predicted $3-\sigma$ intervals begin to slightly diverge for the y- and z-axes at approximately $\omega > 5\ \text {deg./s}$. This indicates that (9) is a reasonable approximation for the error covariance, as expected. The empirical $3-\sigma$ intervals are calculated from the diagonal elements of

10 $$\begin{aligned} {\textbf{R}}_{\omega } = \left( \frac{{\boldsymbol{\Delta \omega \Delta \omega }}^{T}}{N}\right)  \end{aligned}$$

where $\Delta \omega$ represent the angular velocity errors. However, (9) fails when the attitude noise is anisotropic, as evident in Fig. 3. The anisotropic attitude noise is defined as $${\textbf{R}}_{1} = {\textbf{R}}_{2} = \textrm{diag}\left( \begin{bmatrix} 2\times 10^{-3}&2\times 10^{-3}&2\times 10^{-2} \end{bmatrix}\right) \ \text {degree}^{2}$$.

Figure 3 illustrates that the *y*-axis errors begin to spread out at approximately $\omega > 2 \ \text {deg./s}$ causing the predicted $3-\sigma$ intervals derived from (9) to significantly diverge from both the overall trend of the errors and the empirical $3-\sigma$ intervals. Additionally, the *z*-axis errors begin to thin out slightly at $\omega > 5 \ \text {deg./s}$, resulting in further divergence between the predicted and empirical $3-\sigma$ intervals. The errors and the $3-\sigma$ intervals for both types on the *x*-axis remain unaffected. The changes on the *y*- and the *z*-axes errors are caused by projections of the larger *z*-axis error of ${\textbf{q}}_{1}$ projecting onto the *y*-axis of ${\textbf{q}}_{2}$ where we have placed the ${\boldsymbol{\delta }}{\textbf{q}}_{21}$ error rotation. Given this analysis, it is apparent that (9) is insufficient for the anisotropic case and that a new approximation for ${\textbf{R}}_{\omega }$ is needed.

### Improved Angular Velocity Error Covariance Model

The improved approximation of ${\textbf{R}}_{\omega }$ starts with the attitude measurements. Each of these measurements are corrupted by noise which, in turn, corrupts the angular velocity estimate. This degradation is represented as

11 $$\begin{aligned} \widetilde{{\textbf{C}}}_{1I} = {\boldsymbol{\delta }} {\textbf{C}}_{1}{\textbf{C}}_{1I} = \textrm{e}^{{\boldsymbol{\delta \phi }}_{1}^{\wedge }}_{}{\textbf{C}}_{1I}^{} = \left( {\textbf{I}}-{\boldsymbol{\delta \phi }}^{\wedge }_{1}\right) {\textbf{C}}_{1I}^{} \end{aligned}$$

Expanding this formulation to the total rotation leads to

12 $$\begin{aligned} \widetilde{{\textbf{C}}}_{21} = \widetilde{{\textbf{C}}}_{2I}\widetilde{{\textbf{C}}}_{I1} = \widetilde{{\textbf{C}}}_{2I}^{} \widetilde{{\textbf{C}}}_{1I}^{T} = \left( {\textbf{I}}-{\boldsymbol{\delta \phi }}_{2}^{\wedge }\right) {\textbf{C}}_{21}^{}\left( {\textbf{I}}+{\boldsymbol{\delta \phi }}_{1}^{\wedge }\right) \end{aligned}$$

and converting this into axis-angle form leads to

13 $$\begin{aligned} \widetilde{{\boldsymbol{\phi }}}_{21} = \ln \left( \textrm{e}^{{\boldsymbol{\delta \phi }}_{2}^{\wedge }}\textrm{e}^{{\boldsymbol{\phi }}_{21}^{\wedge }}\textrm{e}^{-{\boldsymbol{\delta \phi }}_{1}^{\wedge }}\right) ^{\vee } \end{aligned}$$

Using the Baker-Campbell-Hausdorff approximation [29], and assuming that the measurement errors are small, the combined effect is approximated as

14 $$\begin{aligned} \widetilde{{\boldsymbol{\phi }}}_{21} \approx {\boldsymbol{\phi }}_{21} - {\textbf{J}}_{L}^{-1} \left( {\boldsymbol{\phi }}_{21}^{}\right) {\boldsymbol{\delta \phi }}_{1}^{} + {\textbf{J}}_{R}^{-1} \left( {\boldsymbol{\phi }}_{21}^{}\right) {\boldsymbol{\delta \phi }}_{2}^{} \end{aligned}$$

where ${\textbf{J}}_{R}^{-1}\left( {\boldsymbol{\phi }}_{21}^{}\right)$ and ${\textbf{J}}_{L}^{-1}\left( {\boldsymbol{\phi }}_{21}^{}\right)$ are the right and left inverse Jacobians of SO(3), respectively. These inverses are calculated as

15 $$\begin{aligned} {\textbf{J}}_{R}^{-1} \left( {\boldsymbol{\phi }}_{21}^{}\right)&= \frac{\phi _{21}}{2}\cot \left( \frac{\phi _{21}}{2}\right) {\textbf{I}} + \left[ 1-\frac{\phi _{21}}{2}\cot \left( \frac{\phi _{21}}{2}\right) \right] {\textbf{a}}_{21} {\textbf{a}}_{21}^{T} + \frac{\phi _{21}}{2}{\textbf{a}}_{21}^{\wedge } \end{aligned}$$

16 $$\begin{aligned} {\textbf{J}}_{L}^{-1} \left( {\boldsymbol{\phi }}_{21}^{}\right)&=\frac{\phi _{21}}{2}\cot \left( \frac{\phi _{21}}{2}\right) {\textbf{I}} + \left[ 1-\frac{\phi _{21}}{2}\cot \left( \frac{\phi _{21}}{2}\right) \right] {\textbf{a}}_{21}^{}{\textbf{a}}_{21}^{T}-\frac{\phi _{21}}{2}{\textbf{a}}_{21}^{\wedge } \end{aligned}$$

Inspecting (15) and (16), we can recognize that the inverse Jacobians are transposes of each other, that is, ${\textbf{J}}_{R}^{-1} \left( {\boldsymbol{\phi }}_{21}^{}\right) = {\textbf{J}}_{L}^{-T}\left( {\boldsymbol{\phi }}_{21}^{}\right)$. Thus, we can express the angular velocity error in terms of the errors on the attitude measurements as

17 $$\begin{aligned} \begin{aligned} {\boldsymbol{\delta \omega }} = \widetilde{{\boldsymbol{\omega }}}-{\boldsymbol{\omega }}&= \frac{\widetilde{{\boldsymbol{\phi }}}_{21}-{\boldsymbol{\phi }}_{21}}{\Delta t} = \frac{{\textbf{J}}_{R}^{-1} \left( {\boldsymbol{\phi }}_{21}^{}\right) {\boldsymbol{\delta \phi }}_{2}^{} - {\textbf{J}}_{L}^{-1} \left( {\boldsymbol{\phi }}_{21}^{}\right) {\boldsymbol{\delta \phi }}_{1}^{}}{\Delta t} \\&= \frac{{\textbf{J}}_{R}^{-1} \left( {\boldsymbol{\phi }}_{21}^{}\right) {\boldsymbol{\delta \phi }}_{2}^{} - {\textbf{J}}_{R}^{-T} \left( {\boldsymbol{\phi }}_{21}^{}\right) {\boldsymbol{\delta \phi }}_{1}^{}}{\Delta t} \end{aligned} \end{aligned}$$

The angular velocity covariance is defined in terms of angular velocity errors, ${\boldsymbol{\delta \omega }}$,

18 $$\begin{aligned} {\textbf{R}}_{\omega } = \textrm{E}\left\{ {\boldsymbol{\delta \omega }}{\boldsymbol{\delta \omega }}^{T}\right\} \end{aligned}$$

Expanding this expression and expressing it in terms of the known measurement covariances, and recognizing that the cross-covariance terms between uncorrelated errors are zero, leads to

19 $$\begin{aligned} \begin{aligned} {\textbf{R}}_{\omega }&= \frac{{\textbf{J}}_{R}^{-1} \left( {\boldsymbol{\phi }}_{21}^{}\right) {\textbf{R}}_{2}^{} {\textbf{J}}_{R}^{-T} \left( {\boldsymbol{\phi }}_{21}^{}\right) + {\textbf{J}}_{L}^{-1} \left( {\boldsymbol{\phi }}_{21}^{}\right) {\textbf{R}}_{1}^{} {\textbf{J}}_{L}^{-T} \left( {\boldsymbol{\phi }}_{21}^{}\right) }{\Delta t^{2}} \\&=\frac{{\textbf{J}}_{R}^{-1} \left( {\boldsymbol{\phi }}_{21}^{}\right) {\textbf{R}}_{2}^{} {\textbf{J}}_{R}^{-T} \left( {\boldsymbol{\phi }}_{21}^{}\right) + {\textbf{J}}_{R}^{-T} \left( {\boldsymbol{\phi }}_{21}^{}\right) {\textbf{R}}_{1}^{} {\textbf{J}}_{R}^{-1} \left( {\boldsymbol{\phi }}_{21}^{}\right) }{\Delta t^{2}} \end{aligned} \end{aligned}$$

**Fig. 4:**
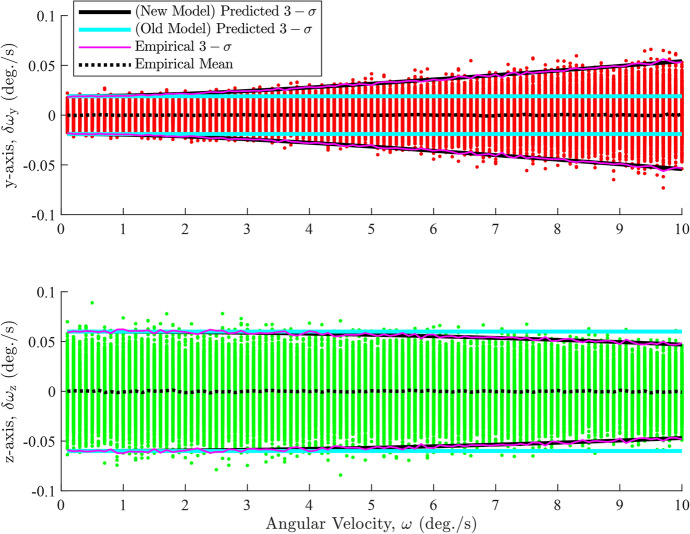
The *y*-axis and *z*-axis angular velocity errors for anisotropic noise with $3-\sigma$ intervals

This improved approximation model addresses the shortcomings of (9), as evident in Fig. 4. This figure shows the *y*- and *z*-axis errors from Fig. 3, but with the predicted $3-\sigma$ intervals derived from (19). The figure demonstrates that the predicted $3-\sigma$ intervals derived from the improved covariance model align much more closely with the trends of the errors and the empirical $3-\sigma$ intervals than those derived from the Jo et al. model (see 9). This demonstrates the overall effectiveness of the new approximation model. A $\chi ^2$ goodness of fit test also showed that the errors have good consistency with the improved model predictions across the studied range of omegas. The *x*-axis errors are not present in Fig. 4 because no changes in the $3-\sigma$ intervals were noticed when using (19).

Given the improved formulation of ${\textbf{R}}_{\omega }$, we can optimize $\Delta t$ for the FD approximation. Generally, we expect a trade-off between using a small and large $\Delta t$. Using a small $\Delta t$ causes ${\boldsymbol{\omega }}$ estimates to be dominated by noise. However, larger $\Delta t$ values lead to estimates that are dominated by latency induced bias. Thus, by optimizing we should be able to find a $\Delta t$ that minimizes both effects and the error covariance.

## Time Step Optimization

The anisotropic covariance model, (19), shows that increasing the time between measurements will decrease the resulting covariance. Provided that $\phi < \pi$, there are no other constraints on the time between measurements. This model, however, relies on the assumption of constant angular velocity; the greater the rate change during the FD period, the larger the inaccuracy in $\widehat{{\boldsymbol{\omega }}}$. Although not strictly true for rotating bodies, (19) can be thought of as calculating the midpoint velocity of the interval $t\in \left[ t_{1}, t_{2}\right]$, but we are generally interested in determining ${\boldsymbol{\omega }}_{2}$, not the midpoint angular velocity. We therefore expect that there will be an optimal choice of $\Delta t$, balancing the effects of noise and bias. This gives an Optimal Finite Difference (OFD) estimate of angular velocity.

This section presents the formulation of the time step optimization for constant angular acceleration motion. The assumptions underlying the derivation are discussed first, followed by the derivation itself. The section concludes with a discussion of the Monte Carlo simulations used to validate the models and formulations developed in this section.

### Assumptions

Before deriving the optimization of $\Delta t$, the underlying assumptions must be discussed. The first major assumption is that the motion being considered is constant angular acceleration. Although constant acceleration maneuvers are not particularly complex, they are encountered in many types of mission. Long rest-to-rest slews under actuator and rate constraints typically begin and end with segments of near constant acceleration. Although more agile maneuvers may be employed in some missions, inertial pointing, constant velocity, and constant angular accelerations encompass a large range of operations.

The next assumption is that the applied noise is anisotropic and modeled as a zero-mean Gaussian random variable. This assumption ensures that the attitude measurements accurately reflect the type of noise typically present in star tracker measurements. Furthermore, by using the anisotropic assumption, we can use the improved angular velocity covariance model, (19), for the optimization derivation, providing an additional means of validating the improved covariance model. The next major assumption is that the noise covariance matrices are diagonal and constant over time, i.e., ${\textbf{R}}_{1} = {\textbf{R}}_{2}$. This assumption is again made to reflect the typical noise characteristics observed in star tracker measurements.

The final assumption is that the axis of rotation, ${\textbf{a}}$, remains constant for both the true and measurement models. In general, attitude motion does not support this assumption as arbitrary torques and gyroscopic coupling can give rise to large changes in the angular velocity. However, during many controlled maneuvers the assumption is not unreasonable, and several factors support this assertion. First, we focus on active control scenarios where we expect to find star trackers in use. Consider the matrix form of Euler’s equations,

20 $$\begin{aligned} {\textbf{J}}\dot{{\boldsymbol{\omega }}}+{\boldsymbol{\omega }}^{\times }{\textbf{J}}{\boldsymbol{\omega }} = {\textbf{M}} \end{aligned}$$

where ${\textbf{J}}$ is the inertia matrix and ${\textbf{M}}$ is the external moment.

Changes in the rotational axis over short timescales can be caused either by agile maneuvering or gyroscopic coupling between axes. In the former case, we can add the additional stipulation that the sampling rate of a star tracker must be sufficient to adequately capture the spacecraft motion. In the latter case, a realistic operational scenario can illustrate the effects of the gyroscopic coupling term. We base this scenario on the reorientation of the X-ray Timing Explorer (XTE) as analyzed by Wie and Lu [30]. Although the example is not recent, the XTE mission had both a) three distinct principal moments of inertia, and b) fast slew requirements. These are the two factors that create strong gyroscopic interactions in (20).

Wie and Lu’s formulation presents a PD-like quaternion-feedback controller with eigenaxis compensation and saturation logic to account for limits on angular rate and allowable reaction wheel torque. The quaternion-feedback controller has the form

21 $$\begin{aligned} {\textbf{u}}=\underset{\sigma }{\text {sat}}\left( {\textbf{K}}\text {sat}\left( {\textbf{P}}{\textbf{q}}_{v}\right) +{\textbf{C}}{\boldsymbol{\omega }}\right) \end{aligned}$$

where ${\textbf{K}}$, ${\textbf{P}}$, and ${\textbf{C}}$ are gain matrices and ${\textbf{q}}_{v}$ is the vector component of the error quaternion.

**Fig. 5:**
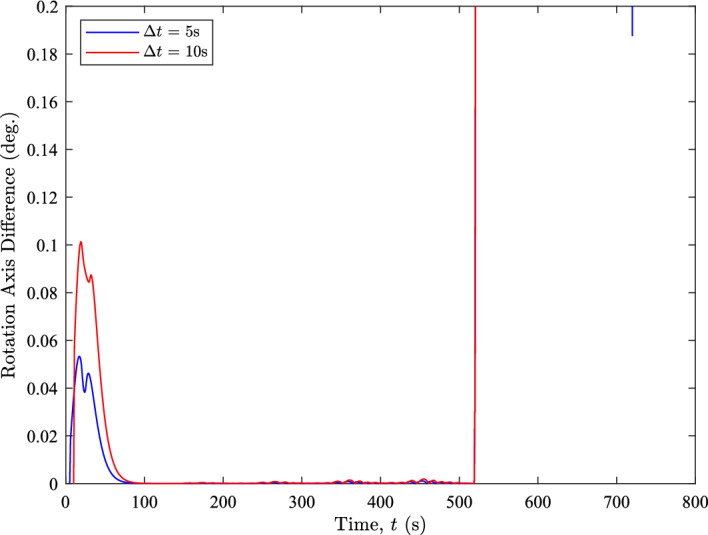
The rotation axis difference of ${\boldsymbol{\omega }}$ throughout the XTE realignment

The inertia matrix used in the example is $${\textbf{J}} = \text {diag}\left( \begin{bmatrix} 6292&5477&2687 \end{bmatrix} \right) \,\text {kg}\cdot \text {m}^{2}$$ with initial orientation $${\textbf{q}}_{v}\left( 0\right) = \begin{bmatrix} 0.2652&0.2652&-0.6930 \end{bmatrix}$$. The nominal scenario completes a $104^{\circ }$ slew in about $9\ \text {minutes}$. Figure 5 shows the angular change in the ${\boldsymbol{\omega }}$ axis for the entire motion over $\Delta t$ values of 5 and 10 s.

The figure demonstrates that there is little change in the rotation axis when using both time steps even with eigenaxis compensation disabled. During the initial acceleration, the system exhibits a maximum axis change of about $0.1^{\circ }$ over a $\Delta t=10\ \text {s}$ but is otherwise almost two orders of magnitude smaller for most of the slew. At the end of the slew, there is a little chatter in the estimated rotation axis as the system comes to rest, but in this regime the angular velocities are near zero. This chatter may require careful control design if the entire spacecraft is operating without rate gyros, but is unlikely to cause problems when used as an internal rate within the star tracker. These observations indicate that, for most portions of the motion where angular velocity estimation occurs, the assumption of a constant axis of rotation is reasonably valid.

**Fig. 6:**
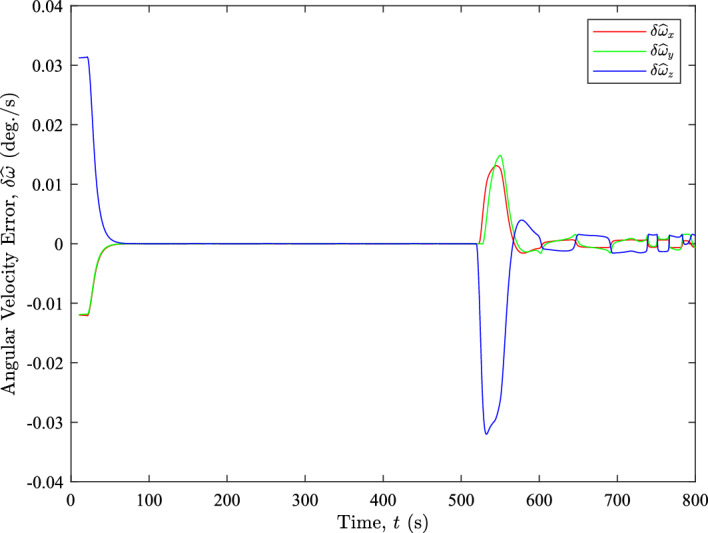
The angular velocity error throughout the XTE realignment

We can further demonstrate that the constant axis of rotation assumption is reasonable by examining how changes in the rotation axis affect the angular velocity error when using the FD method. Figure 6 presents the angular velocity error between the estimated and true angular velocities. The time step used for the estimation is $\Delta t = 10\ \text {s}$.

**Fig. 7:**
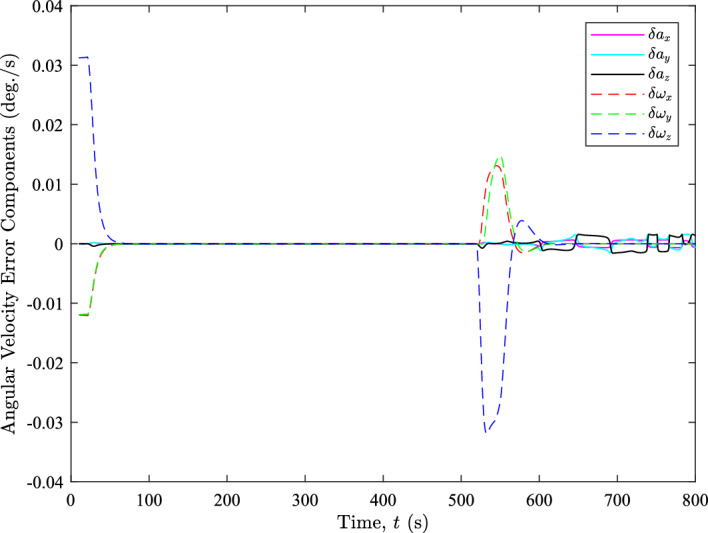
The angular velocity error components throughout the XTE realignment

The figure illustrates that relatively large angular velocity errors occur where differences in the rotation axis are observed, as shown in comparison with Fig. 6. However, these errors are primarily caused by bias errors resulting from acceleration and deceleration during those time intervals. We can verify this by computing the true bias error for the motion, defined as the difference between the average true angular velocity over the $\Delta t = 10\ \text {s}$ window and the true angular velocity at the time of estimation. Figure 7 presents the angular velocity error components for each axis. The error contributions due to the true bias error are denoted as $\delta \omega _{x}$, $\delta \omega _{y}$, and $\delta \omega _{z}$, while those due to rotation axis differences are denoted as $\delta a_{x}$, $\delta a_{y}$, and $\delta a_{z}$. The error contribution by the rotation axis difference is obtained by subtracting the true bias error from the angular velocity error shown in Fig. 6.

Figure 7 clearly show that nearly all of the angular velocity error observed in Fig. 6 is attributable to the bias error across all axes. Although the rotation axis differences contribute slightly to the angular velocity error, their impact is negligible compared to the bias error contributions. These observations indicate that the constant axis of rotation assumption is not perfect but still reasonable. However, these results do indicate that during periods where angular acceleration is present, the bias error becomes quite significant. This suggests that bias correction using angular acceleration would be beneficial, but a thorough discussion of this topic is beyond the scope of the paper. Nevertheless, we have identified it as an area for future work.

### Optimal Time Delay Derivation

With the assumptions established, we can now derive the $\Delta t$ optimization. Under the assumption of a constant axis of rotation, the magnitudes of the angular rotation and angular velocity for a constant angular acceleration motion are

22 $$\begin{aligned} \theta&= \theta _{0} + \omega _{0} t + \frac{1}{2}\alpha t^{2} \end{aligned}$$

23 $$\begin{aligned} \omega&= \omega _{0} + \alpha t \end{aligned}$$

where $\theta _{0}$ and $\omega _{0}$ are the initial angular rotation and velocity, respectively, and $\alpha$ is the angular acceleration. The relative rotation angle between attitude measurements at $t_{1} \text { and } t_{2}$ in axis-angle form about a constant arbitrary axis is

24 $$\begin{aligned} \phi&= \omega _0\Delta t + \frac{1}{2} \alpha \Delta t^{2} \end{aligned}$$

25 $$\begin{aligned} {\textbf{a}}&= \begin{bmatrix} a_{x}&a_{y}&a_{z} \end{bmatrix}^{T} \end{aligned}$$

where $\Delta t$ is the time between measurements. Knowing the axis of rotation allows us to define the relative rotation and true angular velocity vectors as

26 $$\begin{aligned} {\boldsymbol{\phi }}&= \phi {\textbf{a}} \end{aligned}$$

27 $$\begin{aligned} {\boldsymbol{\omega }}&= \omega {\textbf{a}} \end{aligned}$$

Substituting (24) and (25) into (15) and (16) and using those results in (19), we get ${\textbf{R}}_{\omega }$ to be

28 $$\begin{aligned} {\textbf{R}}_{\omega } = \begin{bmatrix} \sigma _{\omega _{x}}^{2}& \sigma _{\omega _{xy}}^{2}& \sigma _{\omega _{xz}}^{2} \\ \sigma _{\omega _{yx}}^{2}& \sigma _{\omega _{y}}^{2}& \sigma _{\omega _{yz}}^{2} \\ \sigma _{\omega _{xz}}^{2}& \sigma _{\omega _{zy}}^{2}& \sigma _{\omega _{z}}^{2} \end{bmatrix} \end{aligned}$$

To minimize the overall angular velocity errors, we focus on the trace of this matrix. The diagonal terms can be expanded in a second order Taylor series approximation in $\Delta t$, leading to

29 $$\begin{aligned} \sigma _{\omega _{x}}^{2}&= \frac{1}{6}\left\{ 2\left( a_{x}^{2}-1\right) \sigma _{x}^{2}+3a_{y}^{2}\sigma _{z}^{2}+3a_{z}^{2}\sigma _{y}^{2}\right\} \omega _{0}^{2} + \frac{2\sigma _{x}^{2}}{ \Delta t^2} \nonumber \\ \sigma _{\omega _{y}}^{2}&= \frac{1}{6}\left\{ 3a_{x}^{2}\sigma _{z}^{2}+2\left( a_{y}^{2}-1\right) \sigma _{y}^{2}+3a_{z}^{2}\sigma _{x}^{2}\right\} \omega _{0}^{2} + \frac{2\sigma _{y}^{2}}{ \Delta t^2} \nonumber \\ \sigma _{\omega _{z}}^{2}&= \frac{1}{6}\left\{ 3a_{x}^{2}\sigma _{y}^{2}+3a_{y}^{2}\sigma _{x}^{2}+2\left( a_{z}^{2}-1\right) \sigma _{z}^{2}\right\} \omega _{0}^{2}+ \frac{2\sigma _{z}^{2}}{ \Delta t^2} \end{aligned}$$

The goal of this optimization is to reduce the effect of noise and systematic errors, (latency induced bias). To do this we examine the expected angular velocity error which is the root sum square (RSS) of the noise and systematic errors,

30 $$\begin{aligned} \overline{\delta \omega }_{x}&= \sqrt{\sigma _{\omega _{x}}^{2} + \delta \omega _{x}^{2}} \nonumber \\ \overline{\delta \omega }_{y}&= \sqrt{\sigma _{\omega _{y}}^{2} + \delta \omega _{y}^{2}} \nonumber \\ \overline{\delta \omega }_{z}&= \sqrt{\sigma _{\omega _{z}}^{2} + \delta \omega _{z}^{2}} \end{aligned}$$

where $\sigma _{\omega _{x}}^{2}$, $\sigma _{\omega _{y}}^{2}$ and $\sigma _{\omega _{z}}^{2}$ are defined in (29) and $\delta \omega _{x}^{2}$, $\delta \omega _{y}^{2}$ and $\delta \omega _{z}^{2}$ are the bias error terms. In practical terms the expected angular velocity errors represent the standard deviations of the angular velocity errors. The bias error terms are calculated by subtracting the true angular velocity from the estimate.

31 $$\begin{aligned} {\boldsymbol{\delta \omega }} = \widehat{{\boldsymbol{\omega }}}-{\boldsymbol{\omega }} = \frac{{\boldsymbol{\phi }}}{\Delta t}-{\boldsymbol{\omega }} \end{aligned}$$

To minimize noise and systematic errors, we must consider the total expected angular velocity error which is

32 $$\begin{aligned} \overline{\delta \omega }_{\textrm{Total}} = \sqrt{\overline{\delta \omega }_{x}^{2}+\overline{\delta \omega }_{y}^{2} + \overline{\delta \omega }_{z}^{2}} \end{aligned}$$

$\overline{\delta \omega }_{\textrm{Total}}$ also represents the standard deviation of the total angular velocity error. Thus, by minimizing (32) we can find the optimal $\Delta t$ that reduce both effects. For simplicity, we frame this optimization in terms of minimizing $\overline{\delta \omega }_{\textrm{Total}}^{2}$ as it is the same as minimizing $\overline{\delta \omega }_{\textrm{Total}}$. Taking the derivative of $\mathrm {\overline{\delta \omega }_{Total}^{2}}$ and solving for $\Delta t$ we get the optimal $\Delta t$

33 $$\begin{aligned} \Delta t_{\textrm{opt}} = \left( \frac{8\sigma _{x}^{2}+8\sigma _{y}^{2}+8\sigma _{z}^{2}}{a_{x}^{2}\alpha ^{2}+a_{y}^{2}\alpha ^{2}+a_{z}^{2}\alpha ^{2}}\right) ^{\frac{1}{4}} = \left( \frac{4\,\textrm{tr}\left( {\textbf{R}}_{1} + {\textbf{R}}_{2}\right) }{\alpha ^{2}}\right) ^{\frac{1}{4}} \end{aligned}$$

Thus, $\Delta t_{\textrm{opt}}$ is simply a function of the attitude noise and the constant angular acceleration. Knowing these parameters means that we can predict the optimal time step and the corresponding minimum expected angular velocity error.

### Monte Carlo Simulations

To validate that the improved covariance model (19), the expected angular velocity error Eqs. (30) and (32), and the optimal time step expression (33) accurately model their respective quantities, a Monte Carlo simulation was conducted. The simulation estimates the angular velocity and records the errors between the true and estimated angular velocities for a given $\Delta t$. These recorded errors are then used to compute the empirical expected angular velocity errors, which serve to validate the covariance models and the $\Delta t$ optimization.

The simulation begins by generating a set of angular rotations and angular velocities using (22) and (23), based on the parameters of the constant angular acceleration motion, for time *t* ranging from 0 to 25 s. These angular rotations are then converted to quaternion representation, yielding the true attitudes. The angular velocities are converted into vector form using the rotation axis, yielding the true angular velocities.

Next, two true attitude measurements are selected such that time between measurements is $\Delta t$. Once these two true attitude measurements are selected, noise is added to each of them. This is accomplished by randomly generating error rotations for each axis from a normal distribution, denoted as $\delta \theta _{x}$, $\delta \theta _{y}$, and $\delta \theta _{z}$, respectively. The distributions of $\delta \theta _{x}$, $\delta \theta _{y}$, and $\delta \theta _{z}$ are given as

34 $$\begin{aligned} \delta \theta _{x} \sim \mathcal {N}(0,\sigma _{x}),\ \delta \theta _{y} \sim \mathcal {N}(0,\sigma _{y}),\ \delta \theta _{z} \sim \mathcal {N}(0,\sigma _{z}) \end{aligned}$$

The standard deviation values of the normal distribution are defined by the noise covariance matrix, $${\textbf{R}}_{\textrm{1}} = {\textbf{R}}_{\textrm{2}} = \textrm{diag}\left( \begin{bmatrix} \sigma _{x}^2&\sigma _{y}^2&\sigma _{z}^2 \end{bmatrix}\right) \ \text {degree}^{2}$$. After the random error rotations are generated, they are used to compute the error DCMs corresponding to a *z*-*y*-*x* rotation sequence, given as

35 $$\begin{aligned} {\boldsymbol{\delta }}{\textbf{C}} = {\textbf{C}}_{z}(\delta \theta _{z}){\textbf{C}}_{y}(\delta \theta _{y}){\textbf{C}}_{x}(\delta \theta _{x}) \end{aligned}$$

where ${\textbf{C}}_{z}(\delta \theta _{z})$, ${\textbf{C}}_{y}(\delta \theta _{y})$, and ${\textbf{C}}_{x}(\delta \theta _{x})$ denote the principal axis rotation matrices.

The error DCMs are converted into quaternion form referred to as the error quaternions, ${\boldsymbol{\delta }}{\textbf{q}}$. The error quaternions are then quaternion multiplied with the two true attitude quaternions, as defined in (2), to yield the measured attitude quaternions. The relative quaternion is found using (3) and then converted into axis-angle form. The angular velocity is estimated using (6). The error, ${\boldsymbol{\delta \omega }}$, between the true angular velocity at the estimation time and the estimate is recorded. This entire process of adding noise, estimating the angular velocity, and recording the error is repeated 1000 times for each tested $\Delta t$ value.

Finally, the empirical expected angular velocity errors, $\mathrm {\overline{\delta \omega }_{x}}$, $\mathrm {\overline{\delta \omega }_{y}}$, and $\mathrm {\overline{\delta \omega }_{z}}$, for each $\Delta t$ are calculated using the angular velocity errors, ${\boldsymbol{\delta \omega }}$, using

36 $$\begin{aligned} \overline{{\boldsymbol{\delta \omega }}}_{\textrm{Empirical}}&= \begin{bmatrix} \overline{\delta \omega }_{x}&\overline{\delta \omega }_{y}&\overline{\delta \omega }_{z} \end{bmatrix}^{T} =\left[ \textrm{diag}\left( \frac{{\boldsymbol{\delta \omega \delta \omega }}^{T}}{N}\right) \right] ^{\frac{1}{2}} \end{aligned}$$

where $N = 1000$ is the number of trials of the Monte Carlo simulation.

To validate the $\Delta t$ optimization models and formulations, the Monte Carlo simulations considered two constant angular acceleration scenarios. The first scenario involves a pure *x*-axis rotation, defined by a = [1 0 0]^*T*^. The second scenario involves an arbitrary axis of rotation defined by a = [0.6519 0.4632 0.6004]. Apart from these differences, both simulation cases share the same parameters, which are:

- Attitude noise: R_1_ = R_2_ = diag $$\left( \begin{bmatrix} 2\times 10^{-3}&2\times 10^{-3}&2\times 10^{-2} \end{bmatrix}\right)$$ degree^2^
- Initial angular velocity: $\omega _{0} = 1 \ \text {deg./s}$
- Constant angular acceleration: $\alpha = 0.01\ \text {deg./s}^{2}$
- Tested $$\Delta t$$ range: $$1-25 \ \text {s}$$
- Sampling Frequency: 1 Hz

## Results and Discussion

This section analyzes the results of simulations that verify the formulations derived in Sects. 2 and 3. We examine the improved ${\textbf{R}}_{\omega }$ model for the constant angular velocity case with isotropic noise and the constant angular acceleration scenario. We also validate the $\Delta t$ optimization and explore how changing the sampling rate of the measurements affects $\overline{\delta \omega }_{\textrm{Total}}$ and $\Delta t_{\textrm{opt}}$. Lastly, we compare the effectiveness of the FD method with $\Delta t_{\textrm{opt}}$ against a multiplicative Extended Kalman filter (MEKF).

### Constant Angular Velocity

**Fig. 8:**
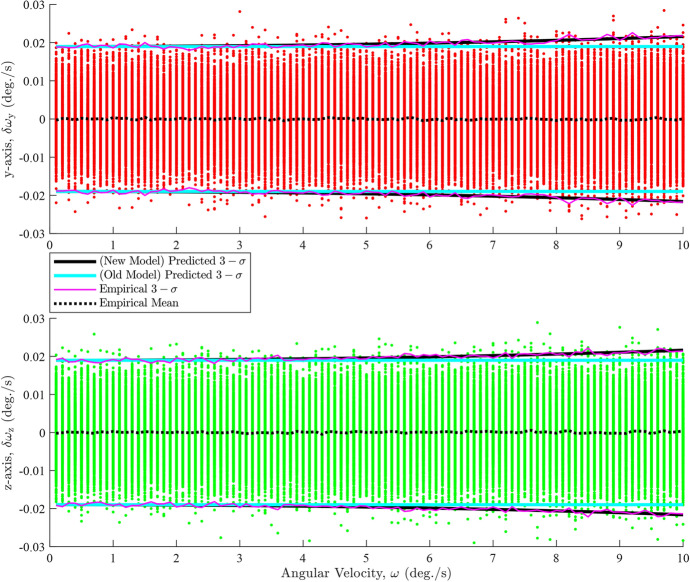
The *y*-axis and *z*-axis angular velocity errors for isotropic noise with $3-\sigma$ intervals

The primary focus of (19) is to approximate ${\textbf{R}}_{\omega }$ in the presence of anisotropic attitude noise. This approximation is also effective for isotropic attitude noise. Figure 8 shows the *y*-axis and *z*-axis errors of Fig. 2 but also with the $3-\sigma$ intervals from (19).

The figure shows that the predicted $3-\sigma$ intervals from (19) align much more closely with the empirical $3-\sigma$ intervals than those derived from (9). A $\chi ^{2}$ goodness of fit test also reinforces this observation, as the errors are far more consistent with the covariances predicted by the new model for the studied ${\boldsymbol{\omega }}$ values than with those calculated from the old model, (9). Additionally, there is no change in the $3-\sigma$ intervals for the *x*-axis errors when using (19).

### Constant Angular Acceleration

We have shown that the improved angular velocity error covariance model is highly accurate for the constant angular velocity scenario. The next step is to examine the effectiveness of this improved covariance model, along with the models of the expected angular velocity errors (see (30) and (32)), for the constant angular acceleration case. The *x*-axis rotation results are discussed first followed by the arbitrary axis of rotation results.

**Fig. 9:**
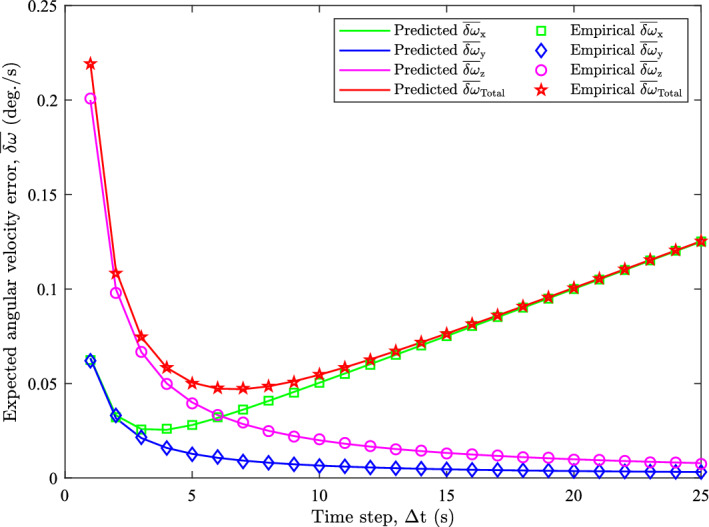
Predicted and empirical $\overline{\delta \omega }$ values for $\alpha = 0.01 \ \text {deg./s}^2$ (*x*-axis of rotation)

The results of the simulation and prediction calculations for the *x*-axis rotation scenario is presented in Fig. 9. This figure illustrates that the predicted $\overline{\delta \omega }$ for all three axes and the total closely following the corresponding empirical quantities. This indicates that the improved ${\textbf{R}}_{\omega }$ approximation, (19), and the expected angular velocity error formulas, (30) and (32), are accurate predictions because there are no obvious deviations between the predicted and empirical curves. Taking the difference between the empirical and predicted quantities we find that the differences are small being less than 8.2 × 10^−4^ deg./s for $\Delta t \ge 3 \ \text {s}$.

**Fig. 10:**
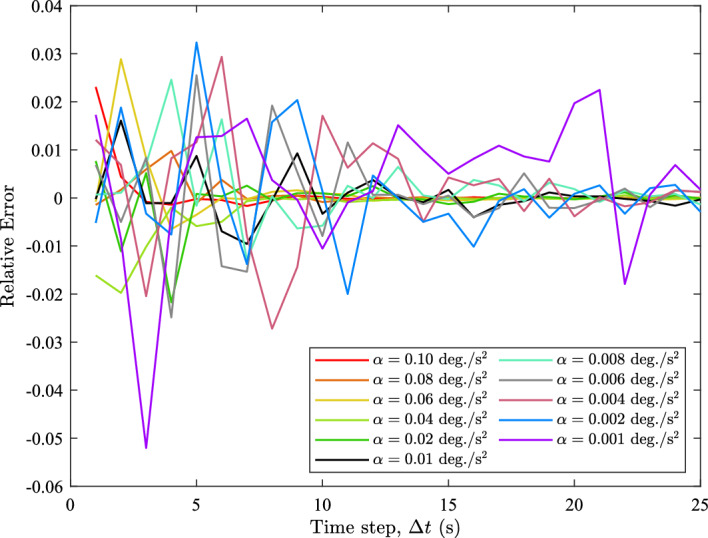
The relative errors between the empirical and predicted $\overline{\delta \omega }$ for all $\alpha$ values tested

Other $\alpha$ values ranging from 0.10 to 0.001 deg./s^2^ were also tested and their results are similar to the outcomes presented so far. Figure 10 shows the relative error between the empirical and predicted $\overline{\delta \omega }_{\textrm{Total}}$ for all $\alpha$ values, i.e., the difference between the empirical and predicted $\overline{\delta \omega }_{\textrm{Total}}$, normalized by the magnitude of the empirical $\overline{\delta \omega }_{\textrm{Total}}$. Figure 10 clearly shows that the relative error is fairly small being within 0.06 from an absolute sense. This illustrates that the predictions of the empirical quantities are quite accurate. We also notice that this observation applies for all $\alpha$ values, which demonstrates that (19), (30), and (32) are robust enough to handle different accelerations.

The analysis of Fig. 10 is further supported by the root mean square errors (RMSE) between the predicted and empirical $\overline{\delta \omega }$ quantities, which are tabulated in Table 1. This table demonstrates that the RMSE values for all angular accelerations and $\overline{\delta \omega }$ quantities are quite small, being $12.8\times 10^{-4}\ \text {deg./s}$ or smaller. This further demonstrates that (19), (30), and (32) accurately models the behaviors of the angular velocity errors.

**Table 1:** The RMSE of $\overline{\delta \omega }$ at each $\alpha$ (*x*-axis rotation)

$\alpha$ (deg./s^2^)	$\overline{\delta \omega }_{\textrm{x}}$ RMSE (× 10^−4^ deg./s)	$\overline{\delta \omega }_{\textrm{y}}$ RMSE (× 10^−4^ deg./s)	$\overline{\delta \omega }_{\textrm{z}}$ RMSE (× 10^−4^ deg./s)	$\overline{\delta \omega }_{\textrm{Total}}$ RMSE (× 10^−4^ deg./s)
0.10	3.09	2.26	11.6	10.4
0.08	5.08	1.72	5.84	4.35
0.06	6.72	4.24	9.64	7.74
0.04	6.80	3.55	11.9	9.06
0.02	4.35	1.85	6.90	5.34
0.01	3.51	4.21	5.34	4.03
0.008	1.95	2.48	4.45	3.74
0.006	2.80	4.41	6.37	5.64
0.004	5.63	2.08	7.99	7.23
0.002	1.96	2.96	6.85	5.85
0.001	4.24	3.80	12.8	11.3

**Fig. 11:**
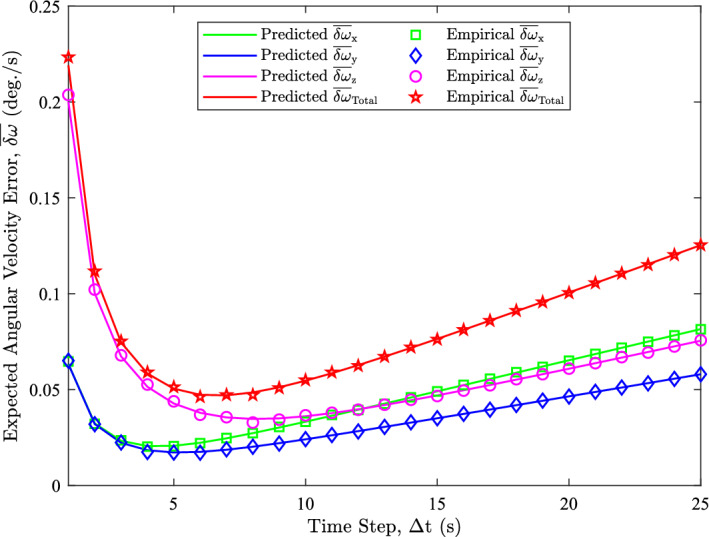
Predicted and empirical $\overline{\delta \omega }$ values for $\alpha = 0.01 \ \text {deg./s}^2$ (arbitrary rotation axis)

We have shown that (19), (30), and (32) accurately models the angular velocity error behaviors for a simple *x*-axis rotation. However, to ensure that the developed models are effective, we need to investigate the arbitrary axis of rotation case. Figure 11 presents the empirical and predicted $\overline{\delta \omega }$ quantities for this scenario.

Figure 11 shows that the predicted and empirical $\overline{\delta \omega }$ quantities closely align. This demonstrates that the improved ${\textbf{R}}_{\omega }$ model and the expected angular velocity error equations accurately model the expected angular velocity errors, as there are no apparent deviations between the quantities. Consequently, we can conclude that (19), (30), and (32) remain effective even for arbitrary rotation axes. This conclusion is further supported by the fact that the analysis of Fig. 11 is consistent with that of Fig. 9.

The RMSE values between the predicted and empirical $\overline{\delta \omega }$ quantities are tabulated in Table 2. It is evident that the RMSE values are small, further confirming that (19), (30), and (32) accurately model the angular velocity error behavior. Comparing these values with the RMSE results for the *x*-axis rotation (Table 1) shows that the RMSE values in both cases are of a similar order of magnitude. This consistency demonstrates that the expected angular velocity error models remain effective.

**Table 2:** The RMSE of $\overline{\delta \omega }$ at each $\alpha$ (arbitrary axis of rotation)

$\alpha$ (deg./s^2^)	$\overline{\delta \omega }_{\textrm{x}}$ RMSE (× 10^−4^ deg./s)	$\overline{\delta \omega }_{\textrm{y}}$ RMSE (× 10^−4^ deg./s)	$\overline{\delta \omega }_{\textrm{z}}$ RMSE (× 10^−4^ deg./s)	$\overline{\delta \omega }_{\textrm{Total}}$ RMSE (× 10^−4^ deg./s)
0.10	2.13	4.14	10.5	7.11
0.08	5.44	5.20	13.2	13.5
0.06	4.93	3.47	7.56	4.47
0.04	3.63	5.75	11.5	8.16
0.02	6.19	5.09	8.19	6.61
0.01	3.22	4.19	10.2	10.3
0.008	3.44	2.96	5.97	4.81
0.006	4.01	5.31	11.6	9.36
0.004	4.05	4.06	16.3	13.2
0.002	1.61	2.97	23.7	21.0
0.001	3.26	9.26	7.32	9.58

### Optimal Time Step Validation

The previous results showed that the improved covariance model and the expected angular velocity error equations are effective at predicting the empirical quantities. Given this, we can show that the $\Delta t$ optimization, (33), is effective.

Figure 9 shows that the minima for the predicted $\overline{\delta \omega }_{\textrm{Total}}$ curve is at $\Delta t = 7 \ \text {s}$ which is the optimal value. Using the $\Delta t_{\textrm{opt}}$ formula, (33), we obtain a $\Delta t_{\textrm{opt}} = 6.62 \ \text {s}$. This value must be discretized to match the sampling rate of the measurements which for this trial is $1 \ \text {Hz}$. So, testing the two adjacent samples closest to $\Delta t_{\textrm{opt}} = 6.62 \ \text {s}$ using (32) we find that $\Delta t_{\textrm{opt}} = 7 \ \text {s}$ produces the smallest error. This matches the observations of Fig. 9, showing the validity of (33). Additionally, the discretized $\Delta t_{\textrm{opt}}$ matches the $\Delta t$ of the empirical $\overline{\delta \omega }_{\textrm{Total}}$ minima in Fig. 9. This also shows the accuracy of predicting $\Delta t_{\textrm{opt}}$.

Figure 9 also illustrates the effects of noise and systematic errors as the $\overline{\delta \omega }_{\textrm{Total}}$ curve closely follows the $\overline{\delta \omega }_{z}$ curve for $\Delta t \le 3 \ \text {s}$. This shows that noise is more dominant at smaller $\Delta t$ values. At $\Delta t \ge 10 \ \textrm{s}$ we see that latency induced bias is more dominant as $\overline{\delta \omega }_{\textrm{Total}}$ closely follows $\overline{\delta \omega }_{x}$. Additionally, we see that the minima of $\overline{\delta \omega }_{\textrm{Total}}$ occurs just after the $\overline{\delta \omega }_{x}$ and $\overline{\delta \omega }_{z}$ curves intersect which shows that $\Delta t_{\textrm{opt}}$ is the minimization of noise and systematic errors.

These experiments also show that the time step optimization is effective for the arbitrary rotation axes case. Because the $\Delta t_{\textrm{opt}}$ formula does not depend on the axis of rotation, the sampling strategy can be formulated knowing only the angular acceleration and the noise characteristics of the sensor. This implies that, for the same noise characteristics and angular acceleration, the optimal time step should remain unchanged. Since the arbitrary axis of rotation simulation used the same angular acceleration and noise parameters, the discretized optimal time step should also be $\Delta t_{\textrm{opt}} = 7 \ \text {s}$. Figure 11 shows that the minimum predicted $\overline{\delta \omega }_{\textrm{Total}}$ occurs at $\Delta t = 7\ \text {s}$, as expected. This confirms that (33) effectively predicts the optimal time step that minimizes the total expected angular velocity error.

The resultant $\Delta t_{\textrm{opt}}$ quantities and their corresponding minimum $\overline{\delta \omega }_{\textrm{Total}}$ for all $\alpha$ values are tabulated in Table 3. The table demonstrates that as $\alpha$ decreases $\Delta t_{\textrm{opt}}$ increases and the minimum $\overline{\delta \omega }_{\textrm{Total}}$ decreases because as $\alpha$ decreases the bias error reduces which shrinks the overall error and increases $\Delta t_{\textrm{opt}}$.

**Table 3:** The optimal $\Delta t$ and corresponding minimum $\overline{\delta \omega }_{\textrm{Total}}$ for each $\alpha$

$\alpha$ (deg/s$^2$)	Predicted $\Delta t_{\textrm{opt}}$ (s)	Discretized $\Delta t_{\textrm{opt}}$ (s)	Minimum $\overline{\delta \omega }_{\textrm{Total}}$ ($\times 10^{-3}$ deg/s)
0.10	2.09	2	148
0.08	2.34	2	136
0.06	2.70	3	116
0.04	3.31	3	94.5
0.02	4.68	5	66.5
0.01	6.62	7	47.0
0.008	7.40	7	42.0
0.006	8.55	9	36.4
0.004	10.47	10	29.7
0.002	14.80	15	21.0
0.001	20.93	21	14.8

### Sampling Rate Sensitivity

All of the experiments in Sect. 4 employed a $1 \ \text {Hz}$ sample frequency. In theory, if the rate was increased this would lead to more accurate results as there would be more measurements to use. To analyze this, the *x*-axis rotation case of the simulation presented in Sect. 3.3 was done 1000 times for each sampling rate and angular acceleration. Sampling rates of 1–10 Hz were tested.

**Fig. 12:**
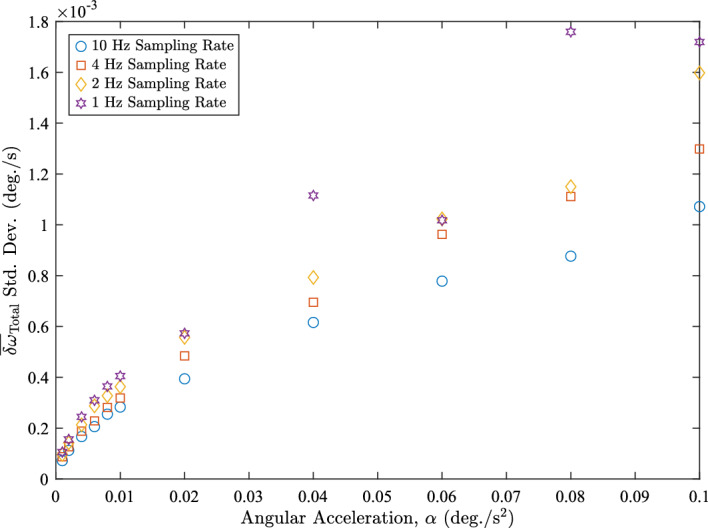
Standard deviations of the empirical minimum $\overline{\delta \omega }_{\textrm{Total}}$ for each sampling rate

Figure 12 shows the standard deviations of the empirical minimum $\overline{\delta \omega }_{\textrm{Total}}$ across the range of sampling rates. For $\alpha \le 0.01 \ \text {deg./s}^{2}$ the standard deviations for each sampling rate are clustered close together. For these angular accelerations, changing the sampling rate does not significantly improve the spread of the minimum $\overline{\delta \omega }_{\textrm{Total}}$, although, we do see some improvement by increasing the sampling rate. However, for $\alpha > 0.01 \ \text {deg./s}^{2}$, we see that the standard deviations have diverged significantly from each other. The $10 \ \text {Hz}$ trials are the best at higher angular accelerations because it is able to capture the changes in the motion more frequently. These observations confirm that having higher sampling rates leads to more accurate angular velocity estimates.

Increasing the sampling rate allows us to select a $\Delta t$ closer to the optimum value. Table 4 shows the predicted and discretized $\Delta t_{\textrm{opt}}$ at the different sampling rates for each $\alpha$. The table demonstrates that over these trials, rounding the optimal $\Delta t$ to the nearest integer number of samples achieves a discrete optimal value.

**Table 4:** Optimal $\Delta t$ for each $\alpha$ and sampling rate

*α* (deg/s_2_)	Predicted $\Delta t_{\textrm{opt}}$ (s)	1 Hz (s)	2 Hz (s)	4 Hz (s)	10 Hz (s)
0.10	2.09	2	2	2	2.10
0.08	2.34	2	2.50	2.25	2.30
0.06	2.70	3	2.50	2.75	2.70
0.04	3.31	3	3.50	3.25	3.30
0.02	4.68	5	4.50	4.75	4.70
0.01	6.62	7	6.50	6.50	6.60
0.008	7.40	7	7.50	7.50	7.40
0.006	8.55	9	8.50	8.50	8.50
0.004	10.47	10	10.50	10.50	10.50
0.002	14.80	15	15	14.75	14.80
0.001	20.93	21	21	21	20.90

### Comparing FD and Recursive Methods

Our optimal finite difference angular velocity estimates show reduced error over naive implementations and our covariance models predict the expected performance better than the isotropic models of Jo et al. [25]. This section expands our evaluation of the OFD solution and contrasts its performance against a more conventional, sequential, angular velocity estimator. The estimator chosen for this comparison is an MEKF based on the works by Critchley-Marrows et al. and Markley et al. [5, 31]. The filter uses discrete nonlinear propagation and quaternion measurements. Implementation details are found in Appendix A.

The scenario for this experiment is the same *x*-axis rotation case as described in Sect. 3.3. The optimized FD estimator is compared to the MEKF over 1000 runs under constant angular acceleration. Two variants of the MEKF are considered; one running at $1\ \text {Hz}$ and another at $10\ \text {Hz}$. The true trajectory was common for each trial and the initial states for the MEKF were generated according to the initial state covariance. The error statistics were calculated at $t=7\,\text {s}$ to allow the MEKF to converge.

For this comparison, the OFD uses the optimal time step based on the true angular acceleration of the scenario, $\Delta t_{\text {opt}} = 7\ \text {s}$, representing the best case performance for the OFD. This was done to see how the OFD method would compare to the MEKF at its best possible performance. Since calculating the optimal time step requires knowledge of the angular acceleration, this raises the question of how that quantity can be obtained in practice. The angular acceleration could potentially be estimated from the available star tracker measurements or provided by the ADCS. A detailed investigation of these possibilities lies beyond the scope of this paper but preliminary experiments have shown promising results.

**Fig. 13:**
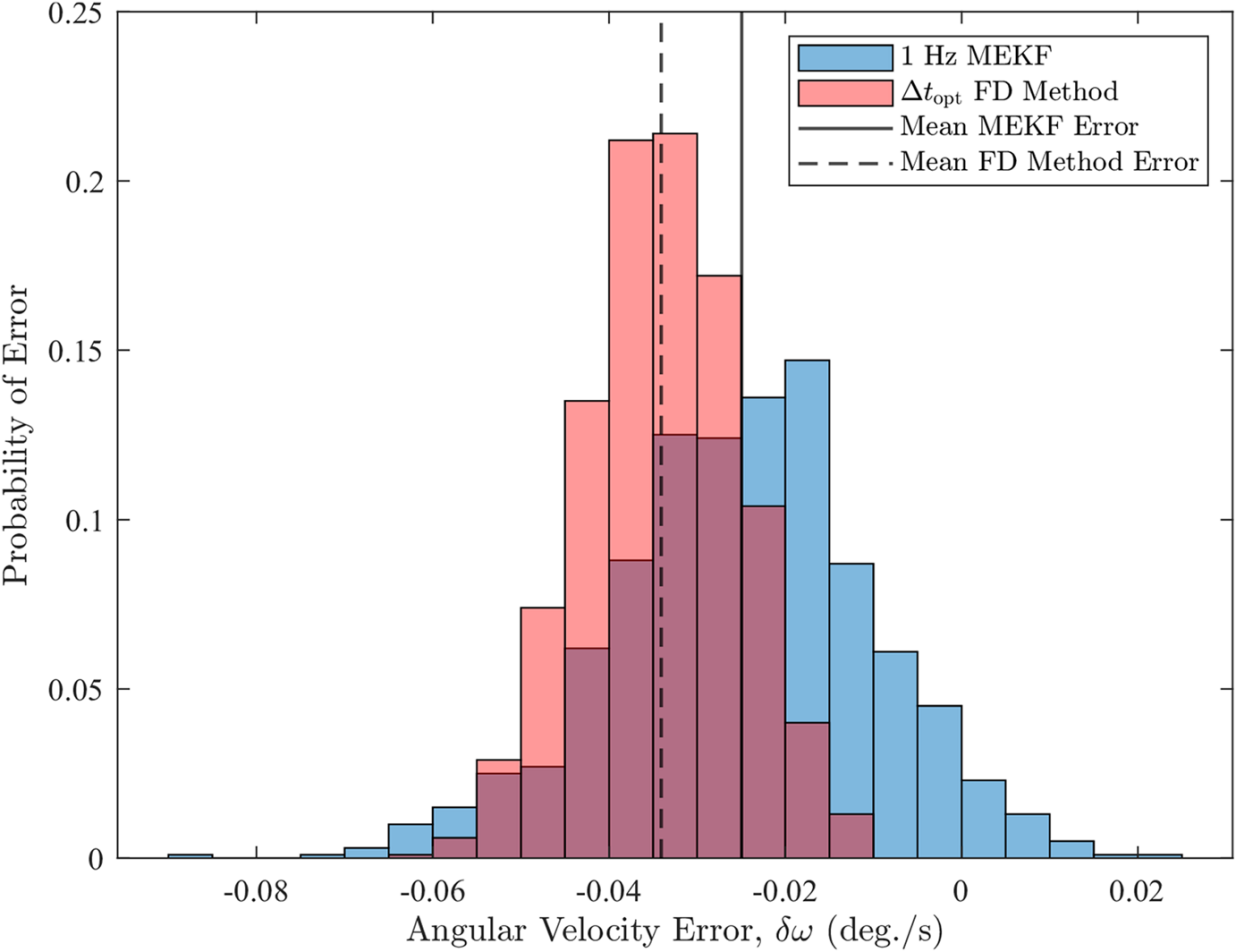
Histograms of the angular velocity errors from the FD method and MEKF with $1 \ \text {Hz}$ sampling rate

**Fig. 14:**
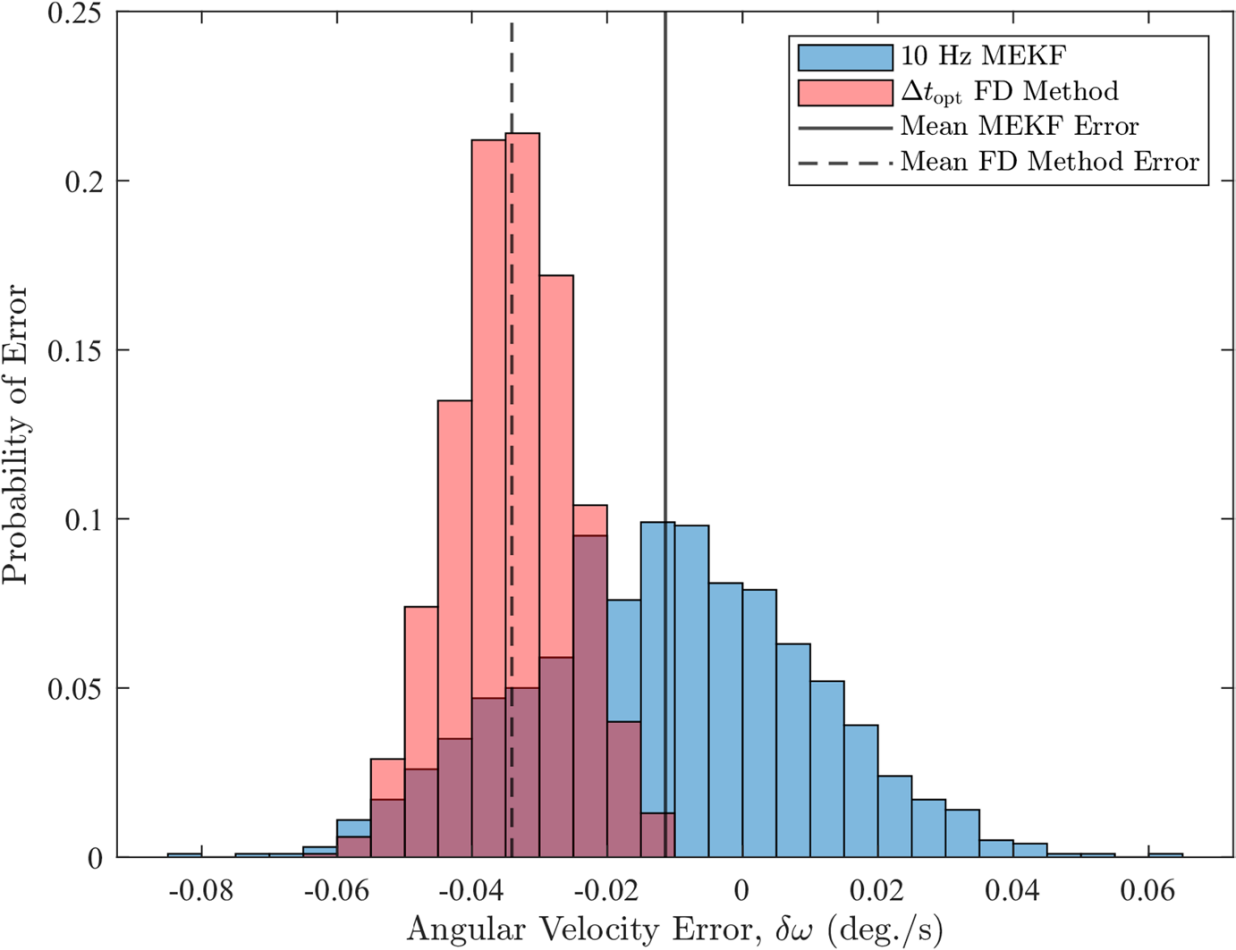
Histograms of the angular velocity errors from the FD method and MEKF with $10 \ \text {Hz}$ sampling rate

Figures 13, 14, and Table 5 show the primary results of the trial. The histograms show the distribution of the errors for each method and the table summarizes the mean error and standard deviations of the histogram data.

**Table 5:** Statistical error results for the FD method and MEKF

Statistic	OFD method (deg./s)	1 Hz MEKF (deg./s)	10 Hz MEKF (deg./s)
Mean error ($\mu$)	−34.1 × 10^−3^	−24.9 × 10^−3^	−11.4 × 10^−3^
Error standard deviation ($\sigma$)	8.79 × 10^−3^	14.8 × 10^−3^	21.1 × 10^−3^

Figures 13 and 14 show that both methods produce biased estimates, but the bias in the OFD estimates is larger than that of the MEKF in both cases. Table 5 demonstrates that the larger bias in the OFD method causes the mean error to be 1.4–3 times larger than the MEKF’s values in absolute terms. These results indicate that the OFD method is generally less accurate than the MEKF as its estimated angular velocities are further away from the true value. Despite this, the OFD approach demonstrates greater precision, as evidenced by its errors being more tightly cluster around the mean error, as shown in Figs. 13 and 14. This is further supported by the fact that standard deviations in the OFD errors are approximately, $40\%$ and $58\%$ smaller than the MEKF’s corresponding values, respectively. Overall, these findings show that the OFD method and the MEKF are comparable because neither technique has a clear advantage over the other. The OFD’s performance suffers due to the latency-induced bias errors but makes up for it by having significantly smaller error standard deviations.

For the OFD method, the bias error arises because the FD approach estimates the midpoint angular velocity of the interval $\left[ t_{1}, t_{2}\right]$ rather than $\boldsymbol{\omega }$ at $t_2$. Although, the optimal time step minimizes this bias error, along with the noise, it does not eliminate it. As a result, the OFD estimates remain biased, leading to the relatively large mean error compared to the MEKF results. The MEKF technique, on the other hand, estimates the average angular velocity over the time interval, $\Delta t$, that minimizes the error between the observed and predicted attitude measurements during propagation. This introduces bias in the estimated angular velocities, as shown in the MEKF results. The bias can be partially mitigated by increasing the number of sample available to the MEKF, as evident from the 10 Hz mean error. These results make it clear that, for the OFD method to be fully effective and to provide angular velocity estimate at $t_2$ rather than the midpoint, bias correction is needed to improve accuracy. Even the MEKF can benefit from bias correction. However, detailed discussion of bias correction and its effect on the estimated angular velocity is beyond the scope of this paper, but has been identified as a direction for future work.

## Conclusion

This study explores finite difference angular velocity approximations appropriate for use in star tracker processors and other gyro-free systems. Our covariance analysis captures the coupling between angular rate and anisotropic error that prior works have not addressed. These models were validated using Monte Carlo trials and the empirical and predicted errors agree to within a few percent, even at high angular rates. This analytical error model provides attitude control practitioners better insight into the expected performance of their systems.

The second primary contribution of this study extends the analysis of angular velocity error and derives an optimal sampling time, $\Delta t_{\textrm{opt}}$, between attitude measurements. This Optimal Finite Difference approach balances the combined effects of latency and noise. Although derived for continuously variable sample times, the relatively shallow optimum allows some implementation flexibility, particularly if sample times are constrained by fixed star tracker exposure times and update rates. OFD estimates compare favorably with MEKF derived estimates and require less computation and tuning to use effectively.

There are a few practical implementation details that merit further study. Calculation of $\Delta t_{\textrm{opt}}$ depends on knowledge of the system angular acceleration and much of the latency induced errors in the OFD angular velocity estimates could also be corrected with this quantity. Although it might be possible to provide feedforward estimates from the spacecraft control system to the star tracker, the additional interface complexity that would be required may not be justified. We are currently exploring techniques to extend the OFD formulation to explicitly estimate acceleration and velocity together—effectively reframing the analysis to separate the known and unknown components of angular acceleration. We are also investigating bias correction and its effect on improved the overall accuracy of the OFD method.

We recognize that in star tracker processing, gyro-free operation is often desirable, yet angular velocity knowledge is still required. These applications benefit from computationally simple, finite difference rate estimates. Our OFD approach requires only minimal additional processing, yet can significantly improve the errors in these systems.

## Data Availability

No datasets were generated or analysed during the current study.

## Appendix A: MEKF Formulation and Results

This appendix discusses the formulation of the MEKF and the initialization parameters. The MEKF is based on the implementations by Critchley-Marrows et al. and Markley et al. [5, 31]. The overall formulation of the MEKF remains the same as any other EKF. However, the difference lies in the fact that the measurement update is applied to the state error matrix, $\Delta {\textbf{x}}_{k}$, rather than the state matrix, ${\textbf{x}}_{k}$. Another key difference is the reset step after the measurement update. This is where ${\textbf{x}}_{k}$ is updated from the pre-measurement to post-measurement status using the updated $\Delta {\textbf{x}}_{k}$. After the update, $\Delta {\textbf{x}}_{k}$ is reset to a zero value. The equations used in the MEKF are listed in Table 6. Note that the MEKF uses scalar-last format for the quaternion representation. Also, the superscripts “−” and “+” indicate pre-measurement and post-measurement update, respectively.

**Table 6:** MEKF formulation

MEKF stage	Equations
States	**x**_*k*_ = $$ \begin{bmatrix} {\mathbf {\widehat{q}}}&{\boldsymbol{\widehat{\omega }}} \end{bmatrix}^{T}$$ = $$\begin{bmatrix} \widehat{q}_1&\widehat{q}_2&\widehat{q}_3&\widehat{q}_0&\widehat{\omega }_{x}&\widehat{\omega }_{y}&\widehat{\omega }_{z} \end{bmatrix}^{T}$$
	$\Delta$**x**_*k*_ = $$ \begin{bmatrix} \delta {\boldsymbol{\theta }}&\delta {\boldsymbol{\omega }}\end{bmatrix}^{T}$$ = $$\begin{bmatrix} \delta \theta _{x}&\delta \theta _{y}&\delta \theta _{z}&\delta \omega _{x}&\delta \omega _{y}&\delta \omega _{z}\end{bmatrix}^{T}$$
Measurement update	${\textbf{K}}_{k} = {\textbf{P}}_{k}^{-}{\textbf{H}}_{k}^{\textrm{T}} \left[ {\textbf{H}}_{k}^{}{\textbf{P}}_{k}^{-}{\textbf{H}}_{k}^{\textrm{T}}+{\textbf{R}}_{k}^{}\right] ^{-1}$
	$${\textbf{H}}_{k} = \begin{bmatrix} {\textbf{I}}_{3\times 3}&0_{3\times 3}\end{bmatrix}$$
	$$\Delta {\textbf{x}}_{k}^{+} = \Delta {\textbf{x}}_{k}^{-} + {\textbf{K}}_{k}^{} \left[ {\textbf{y}}_{k}^{} -{\textbf{h}}_{k}^{}-{\textbf{H}}_{k}^{} \Delta {\textbf{x}}_{k}^{-}\right] $$
	$${\textbf{P}}_{k}^{+} = \left[ {\textbf{I}}_{6\times 6}^{}-{\textbf{K}}_{k}^{}{\textbf{H}}_{k}^{}\right] {\textbf{P}}_{k}^{-}$$
	${\textbf{y}}_{k} = 2 \frac{\left( {\textbf{q}}_{k}^{}\otimes {\mathbf {\widehat{q}}}_{k}^{-1}\right) _{1:3}}{\left( {\textbf{q}}_{k}^{}\otimes {\mathbf {\widehat{q}}}_{k}^{-1}\right) _{0}}$ ${\textbf{h}}_{k} = 2 \frac{\left( {\mathbf {\widehat{q}}}_{k}^{}\otimes {\mathbf {\widehat{q}}}_{k}^{-1} \right) _{1:3}}{\left( {\mathbf {\widehat{q}}}_{k}^{}\otimes {\mathbf {\widehat{q}}}_{k}^{-1}\right) _{0}}$
Reset	$${\textbf{x}}_{k}^{+} = \begin{bmatrix}{\mathbf {\widehat{q}}}_{k}^{+}&{\boldsymbol{\widehat{\omega }}}_{k}^{+}\end{bmatrix}^{\textrm{T}}$$
	$${\mathbf {\widehat{q}}}_{k}^{*+} = {\mathbf {\widehat{q}}}_{k}^{-} + \frac{1}{2}{\boldsymbol{\Xi }} \left( {\mathbf {\widehat{q}}}_{k}^{-}\right) \delta {\boldsymbol{\theta }}_{k}^{+}$$
	$${\boldsymbol{\Xi }}\left( {\mathbf {\widehat{q}}}_{k}^{-}\right) = \begin{bmatrix} \widehat{q}_{0}^{} {\textbf{I}}_{3\times 3}^{}+{\boldsymbol{\widehat{q}}}_{1:3}^{\times }\\ -{\boldsymbol{\widehat{q}}}_{1:3}^{\textrm{T}} \end{bmatrix}$$
	${\mathbf {\widehat{q}}}_{k}^{+} = \frac{{\mathbf {\widehat{q}}}_{k}^{*+}}{|{\mathbf {\widehat{q}}}_{k}^{*+}|}$ ${\boldsymbol{\widehat{\omega }}}_{k}^{+} = {\boldsymbol{\widehat{\omega }}}_{k}^{-} + \delta {\boldsymbol{\widehat{\omega }}}_{k}^{+}$
Propagation	$\dot{\widehat{{\textbf{q}}}}_{\mathrm {k+1}}^{-} = \frac{1}{2} {\boldsymbol{\Xi }}\left( {\mathbf {\widehat{q}}}_{k}^{+}\right) {\boldsymbol{\widehat{\omega }}}_{k}^{+}$
	$${\boldsymbol{\widehat{\omega }}}_{\mathrm {k+1}}^{-} = {\boldsymbol{\widehat{\omega }}}_{k}^{+}$$
	$${\mathbf {\widehat{P}}}_{\mathrm {k+1}}^{-} = \Phi {\textbf{P}}_{k}^{+}\Phi ^{\textrm{T}}+{\textbf{Q}}$$
	$$\Phi = \begin{bmatrix} \Phi _{\delta {\boldsymbol{\theta }}\delta {\boldsymbol{\theta }}} & \Phi _{\delta {\boldsymbol{ \theta }}\delta {\boldsymbol{\omega }}} \\ 0_{3\times 3} & {\textbf{I}}_{3\times 3} \end{bmatrix}$$
	$\Phi _{\delta {\boldsymbol{\theta }}\delta {\boldsymbol{\theta }}}$ = ${\textbf{I}}_{3\times 3}^{}$ − $\left[ {\boldsymbol{\widehat{\omega }}}_{k}^{+}\right] ^{\times } \frac{\sin {\left( |{\boldsymbol{\widehat{\omega }}}_{k}^{+}|\Delta t \right) }}{|{\boldsymbol{\widehat{\omega }}}_{k}^{+}|}$ + $\left( \left[ {\boldsymbol{\widehat{\omega }}}_{k}^{+} \right] ^{\times }\right) ^{2} \frac{1-\cos {\left( |{\boldsymbol{\widehat{\omega }}}_{k}^{+}|\Delta t \right) }}{|{\boldsymbol{\widehat{\omega }}}_{k}^{+}|^{2}}$
	$$\Phi _{\delta {\boldsymbol{\theta }}\delta {\boldsymbol{\omega }}} $$ = $$ \left[ {\boldsymbol{\widehat{\omega }}}_{k}^{+}\right] ^{\times } $$$$ \frac{1-\cos {\left( |{\boldsymbol{\widehat{\omega }}}_{k}^{+}|\Delta t\right) }}{|{\boldsymbol{\widehat{\omega }}}_{k}^{+}|^{2}} $$+ $${\textbf{I}}_{3\times 3}^{}\Delta t - \left( \left[ {\boldsymbol{\widehat{\omega }}}_{k}^{+}\right] ^{\times } \right) ^{2} \frac{|{\boldsymbol{\widehat{\omega }}}_{k}^{+}|\Delta t-\sin {\left( |{\boldsymbol{\widehat{\omega }}}_{k}^{+}|\Delta t\right) }}{|{\boldsymbol{\widehat{\omega }}}_{k}^{+}|^{3}}$$

The simulation scenario for the MKEF is the same as the one described in Sect. 4.2 with the same parameters. To initialize the MEKF the first guess of the state, ${\textbf {x}}_{0}$, and covariance, ${\textbf {P}}_{0}$, is

- $${\textbf {x}}_{0} = \begin{bmatrix} 0&0&0&1&0.0131&0&0 \end{bmatrix}^{T}$$
- $${\textbf {P}}_{0} = \left( 8.7266\times 10^{-4}\right) ^{2} {\textbf {I}}_{6\times 6}$$

The initial guess of the state corresponds to a starting rotation of zero degrees about all axes with an initial angular speed of $0.75 \ \text {deg./s}$ on the *x*-axis. These starting values were chosen such that the MEKF had a relatively poor initial guess of the angular velocity. They were chosen to show how quickly the MEKF can converge on a solution. The process noise covariance is ${\textbf {Q}} = \left( 4.3633\times 10^{-4}\right) ^{2} {\textbf {I}}_{6\times 6}$.
